# Efficacy of Rivaroxaban Use in Solid Tumour Malignancy: Experience from a Tertiary Care Cancer Centre

**DOI:** 10.31557/APJCP.2021.22.11.3601

**Published:** 2021-11

**Authors:** Azmi Nor Mohd Farez Ahmat, Sharifa Ezat Wan Puteh, Suhana Yusak

**Affiliations:** 1 *Department of Community Health, Faculty of Medicine, Universiti Kebangsaan Malaysia Medical Centre, Kuala Lumpur, Malaysia. *; 2 *Department of Pharmacy, National Cancer Institute, Putrajaya, Malaysia. *; 3 *Department of Radiotherapy & Oncology, National Cancer Institute, Putrajaya, Malaysia. *

**Keywords:** Cancer, associated thrombosis, rivaroxaban, factor Xa inhibitor, anticoagulants, real-world

## Abstract

**Objective:**

Cancer-associated venous thromboembolism (CAT) is a common disease or complication which is associated with reduced survival and incurring a substantial health-care cost. Low molecular weight heparin (LMWH) remained the gold standard treatment option available. Direct oral anticoagulants (DOACs) have recently become more popular in the guidelines, they are still few and inconsistent across the current literature. The aim of this study was to evaluate rivaroxaban in treatment of CAT.

**Methods:**

In this prospective real-world study, we recruited and followed up patients diagnosed with CAT treated with rivaroxaban or standard of care as a control for 12 months or until death. Baseline characteristics were collected at the study entry. The primary outcomes were recurrent DVT or PE and death within 12 months after treatment initiation. Safety outcomes were composite outcomes of major and minor bleeding.

**Results:**

A total of 80 patients confirm CAT with radiological imaging were recruited; 39 patients were evaluated in the control arm and 41 patients in the rivaroxaban arm. The 12 months cumulative CAT recurrence rate was 46.2% in control and 39% in rivaroxaban (p=0.519). The 12-month death was not a statistically significant difference between both arms (20.5% vs. 31.7%, p=0.255). The cumulative rate of composite safety outcomes was similar in both groups (17.9% vs. 12.2%, p=0.471).

**Conclusion:**

The result of this small but important real-world evidence proofs that rivaroxaban is an effective and safe alternative to the standard of care for CAT in Malaysia’s cancer population.

## Introduction

Venous thromboembolism (VTE) is a fairly common disease or complication which is associated with reduced survival and incurring substantial health-care cost (Heit 2015). VTE is defined as the formation of blood clots in the veins. However, in cancer patient’s cancer associated VTE (CAT) can occur in both artery and vein (Kim et al., 2020). Two most common forms of VTE are deep vein thrombosis (DVT) and pulmonary embolism (PE). The estimated number of incidence of VTE in the general population is approximately 1-2/1000 of the population (Ay et al., 2017). This can be substantially underestimated in cancer patients.

Cancer associated VTE (CAT) poses a significant clinical problem in patients with solid tumour malignancy. Nearly all patients with active malignancy demonstrate some degree of activation of coagulation resulting in a hypercoagulable state (Goldhaber and Bounameaux, 2012). Besides that, interaction among cancer cells, host cells, venous stasis, vessel wall injury, and cancer treatment itself has been associated with CAT (Ay et al., 2017). It significantly causes morbidity resulting due to hospitalisation and anti-coagulant use as well as bleeding complications, recurrence, and cancer treatment delays. More significantly, CAT is also associated with higher mortality rate among cancer patients (Ay et al., 2017; Goldhaber and Bournameaux, 2012). 

Even though there has been extensive advancement in the management of CAT, low molecular weight heparin (LMWH) remained gold standard treatment option available. Recently, direct oral anticoagulant (DOAC) which offers patients a more preferred oral route and requires almost no laboratory monitoring; have given some hope for a less invasive treatment option.

The use of this drug particularly rivaroxaban has been ideally proven in the SELECT-D trial. In the randomized SELECT-D trial, 406 patients were given rivaroxaban and dalteparin respectively in a control environment and result were compared (Young et al., 2018). The result of this highly controlled randomized trial which had strict inclusion and exclusion criteria may not always mimic real life clinical practice (Stang, 2011). Routinely, we saw more patients that are normally excluded from clinical trials. Lately, American Society of Clinical Oncology (ASCO) and National Comprehensive Cancer Network (NCCN) have revised their recommendations and have added the use of rivaroxaban and edoxaban for management of CAT (Key et al., 2019; NCCN Guidelines, 2021). 

Although the recommendations for the use of DOACs has recently become more popular in the guidelines, they are still few and inconsistent across the current literature. Sole decision to use based on scientifically controls RCT with vigorous inclusion and exclusion criteria and in the absence of real-world evidence (RWE). We designed this prospective RWE single centre study to investigate the efficacy and safety profile of rivaroxaban over standard of care in patient with CAT.

## Materials and Methods

*Design*

This study was conducted at Malaysia main tertiary cancer centre. Patients were recruited and followed up were done for 12 months or until death whichever came first. All data were collected under RWE condition in National Cancer Institute, Malaysia. Ethical approval was obtained from Universiti Kebangsaan Malaysia Research Ethical Committee (FF-2019-474) and Ministry of Health Medical Research Ethical Committee (NMRR-19-565-45643). 

*Patient population*

Patients were included if they were at least 18 years of age, had a diagnosis of cancer and concurrent radiological diagnosis of CAT. Allocation of treatment was done using simple data registration list. Patients allocated to rivaroxaban received rivaroxaban 15 mg twice-daily for a total of 3 weeks followed by rivaroxaban 20mg once-daily. Patients allocated to standard of care received either enoxaparin twice-daily or fondaparinux once-daily based on body weight. Patients will be excluded if they received anticoagulant less than 30 days and received both comparators for empirical treatment. 

*Outcome*

There are two primary outcomes. The first primary outcome is recurrent DVT or PE within 12 months after treatment initiation confirm with radiological imaging. The second primary outcome is death within 12 months after treatment initiation. 

The secondary outcome was to compare safety of rivaroxaban vs. standard of care in patients with CAT. The composite outcomes of major and minor bleeding were used. The major bleeding was defined as clinically overt if it was associated with a drop in haemoglobin of 2g/dL, required transfusion of 2 units of packed red blood cells, involved critical site bleeding (intracranial, intraspinal, intraocular, retroperitoneal, or pericardial area), or if it contributed to death. Minor bleeding was defined as overt bleeding not meeting the criteria for major bleeding but associated with medical intervention, unscheduled contact with an oncologist, interruption, or discontinuation of anticoagulant treatment, or associated with any discomfort or impairment of activities of daily life (Young et al., 2018; Faqah et al., 2020).

*Study procedure*

Patients who eligible for inclusion and visited study site between November 2019 and May 2020 and consented for the study were recruited. Patients were followed up for 12 month or until death whichever came first. Patient demographic and relevant clinical data were collected during the study period. Patients underwent suitable radiological imaging (US or CT) at the end of study period to access CAT status. Mann-Whitney U test was performed to compare continuous variables. The Chi Square or Fisher exact test was performed to compare categorical variables. Kaplan-Meier estimates were obtained for survival. A Cox model was used to obtain hazard ratios (HR) and associated 95% CIs and to evaluate independent prognostic factor for CAT recurrence and death. All data were analysed using SPSS version 21 with significant level of a = 0.05. 

## Results

*Patient population*

Between November 1st, 2019, to May 31st, 2020; a total of 80 patients were recruited and followed up. Out of the total 80 patients, 41 patients were treated with rivaroxaban and 39 patients were treated with standard of care. In total 66.2% were female with majority of them being of malay ethnicity (63.8%). 

The baseline characteristics, comorbidities were comparable between both treatment arms (Table 1) except for dyslipidaemia. More dyslipidaemia patients were observed in the control arm compared with rivaroxaban (25.6% vs. 7.3%, p=0.035). Most of indication for anticoagulation in our population was deep vein thrombosis which accounted for 57.5% followed by pulmonary embolism (32.5%), and 10% for combination of the above indications. Interestingly, the baseline comorbidities which include diabetes, hypertension, cardiovascular disease, and major surgery within 30days were similar in both arms and mostly low in frequency.

The most common cancer diagnosis was of gynaecological origin which accounts for 30% of the total population. Gynaecological malignancy was predominant in both arms. Majority of our population was diagnosed with CAT during the first 6 months of cancer diagnosis (57.5%). Interestingly, we found that near to 88% of our patient’s is having advance disease. 

*Recurrent CAT and death*

Table 2 shows a comparison of the incidence of CAT recurrence, death within 12 months of diagnosis and bleeding events. Overall, the rate of CAT recurrence was high in control arm compared to rivaroxaban (46.2% vs. 39%, p=0.519). On the other hand, the rate of death within 12 month from CAT diagnosis was high in rivaroxaban group compared to control (31.7% vs. 20.5%, p=0.255). However, both findings were similar statistically. No significant and important prognostic factors were found in the Cox model. 

*Bleeding*

Twelve (12) patients in our study experience bleeding. Majority of them were in the control arm (17.9%) compared to 12.2% in rivaroxaban arm. However, the rate of this composite outcome was similar in both arms (p=0.471).

**Table 1:** Demographic and Baseline Characteristic

Variables	Total (N=80)	Contol (n=39)	Rivaroxaban (n=41)	p-value
Age, years (median,IQR)	57 (15.5)	58 (15)	57 (15.5)	0.704∞
Female sex (n,%)	53 (66.2)	25 (64.1)	28 (68.3)	0.81ᵋ
Weight, kg (median, IQR)	60.9 (17.2)	61.8 (16.2)	60 (19.2)	1.00∞
BMI (median, IQR)	24.7 (7.5)	25.5 (6.2)	23.8 (8.45)	0.236∞
Ethnicity (n,%)				0.39ᵋ
Malay	51 (63.8)	25 (64.1)	26 (63.4)	
Chinese	18 (22.5)	7 (18.0)	11 (26.8)	
Indian	9 (11.3)	5 (12.8)	4 (9.8)	
Others	2 (2.5)	2 (5.1)	0 (0.0)	
Co-morbids (n,%)				
Diabetes mellitus	15 (18.8)	7 (17.9)	8 (19.5)	0.858ᵋ
Hypertension	29 (36.3)	16 (41)	13 (31.7)	0.386ᵋ
Cardiovascular disease	3 (3.8)	1 (2.6)	2 (4.9)	1.00ᵠ
Dyslipidaemia	13 (16.3)	10 (25.6)	3 (7.3)	0.035ᵠ
Others	7 (8.8)	2 (5.1)	5 (12.2)	0.433ᵠ
Cancer diagnosis (n,%)				0.136ᵋ
Colorectal	14 (17.5)	3 (7.7)	11 (26.8)	
Breast	12 (15)	7 (17.9)	5 (12.2)	
Lung	11 (13.8)	8 (20.5)	3 (7.3)	
Gynaecological	24 (30)	10 (25.6)	14 (34.1)	
Germ Cells	11 (13.8)	6 (15.4)	5 (12.2)	
Others	8 (10)	5 (12.8)	3 (7.3)	
Cancer stage (TNM) (n,%)				0.656ᵋ
Stage 1	2 (2.5)	1 (2.6)	1 (2.4)	
Stage 2	7 (8.8)	2 (5.1)	5 (12.2)	
Stage 3	20 (25)	9 (23.1)	11 (26.8)	
Stage 4	51 (63.8)	27 (69.2)	24 (58.5)	
ECOG Performance Status (n,%)				0.633ᵋ
ECOG 0	38 (47.5)	16 (41)	22 (53.7)	
ECOG 1	23 (28.8)	13 (33.3)	10 (24.4)	
ECOG 2	13 (16.3)	7 (18)	6 (14.6)	
ECOG 3	5 (6.3)	3 (7.7)	2 (4.9)	
ECOG 4	1 (1.3)	0	1 (2.4)	
Cancer treatment (n,%)				0.510ᵋ
Chemotherapy	29 (36.3)	14 (35.9)	15 (36.6)	
Radiotherapy	6 (7.5)	4 (10.3)	2 (4.9)	
Targeted therapy	2 (2.5)	2 (5.1)	0 (0.0)	
Hormonal therapy	4 (5)	2 (5.1)	2 (4.9)	
Chemotherapy (n,%)				
Platinum	22 (27.5)	8 (20.5)	14 (34.1)	0.172ᵋ
Taxane	10 (12.5)	5 (12.8)	5 (12.2)	1.00ᵠ
Antimetabolites	13 (16.3)	6 (15.4)	7 (17.1)	0.838ᵋ
Others	6 (7.5)	4 (10.3)	2 (4.9)	0.426ᵠ
CAT (n,%)				
Pulmonary embolism (PE)	26 (32.5)	11 (28.2)	15 (36.6)	0.424ᵋ
Deep vein thrombosis (DVT)	46 (57.5)	25 (64.1)	21 (51.2)	0.529ᵋ
PE and DVT	8 (10)	3 (7.7)	5 (12.2)	0.713ᵠ
Major surgery within 30 days (n,%)	2 (2.5)	1 (2.6)	1 (2.4)	1.00ᵠ
Platelet level, x 10^9^/L (median, IQR)	330.5 (217.8)	339 (232)	310 (218)	0.593∞
Variables	Total (N=80)	Contol (n=39)	Rivaroxaban (n=41)	p-value
Major surgery within 30 days (n,%)	2 (2.5)	1 (2.6)	1 (2.4)	1.00ᵠ
Platelet level, x 109/L (median, IQR)	330.5 (217.8)	339 (232)	310 (218)	0.593∞
Time to first CAT diagnosis (n,%)				0.505ᵋ
< 6 month	46 (57.5)	21 (53.8)	25 (61)	
6 – 12 month	10 (12.5)	4 (10.3)	6 (14.6)	
> 12 month	24 (30)	14 (35.9)	10 (24.4)	

IQR, Interquartile range, ∞, Mann-Whitney U test; ECOG, Eastern Cooperative Oncology Group; ᵋ, Chi-square; ᵠ, Fisher’s Exact Test

**Table 2:** Efficacy and Safety Outcomes

Variables	Total (N=80)	Control (n=39)	Rivaroxaban (n=41)	p-value
Primary outcomes				
CAT recurrence (n,%)	34 (42.5)	18 (46.2)	16 (39)	0.519ᵋ
Death (n,%)	21(26.3)	8 (20.5)	13 (31.7)	0.255ᵋ
Secondary outcomes				
Bleeding (n,%)	12 (15)	7 (17.9)	5 (12.2)	0.471ᵋ

ᵋ, Chi-square; CAT, Cancer-associated venous thrombosis

## Discussion

It is estimated 4 to 20% of patients with cancer will experience CAT during their course of disease (Faqah et al., 2020; Abdol Razak et al., 2018). Majority of them will have CAT at the point of cancer diagnosis or during their first hospitalization and depending on their tumour type (Abdol Razak et al., 2018). In a Danish registry study, patients with CAT had significantly 36% lower one-year survival rate compared with cancer patients without CAT (Sørensen et al., 2000).

In this study, the majority of patients were female (66.2%). These findings seem to be interesting since other studies showed similar percentages of male and female patients (Young et al., 2018; Faqah et al., 2020; Chen et al., 2021). Countless risk factors for CAT have been narrated well in literature (Ay et al., 2017; Abdol Razak et al., 2018). Patient related factors such as female gender has been associated with higher risk of developing CAT (Abdol Razak et al., 2018; Fuentes et al., 2016). Interestingly, we found CAT is more prevalent in gynaecological malignancy compared to others solid tumour. However, this is not so in literatures where colorectal cancer has been associated with higher CAT compared to other malignancy (Young et al., 2018; Faqah et al., 2020; Chen et al., 2021). Our study also showed a higher proportion of patients with stage 3 and 4 based on TNM staging (88%). This value is higher compared to the previous studies (48% and 58%) (Young et al., 2018; Chen et al., 2021). Patients with metastatic disease were found to have a 20-fold increased risk for CAT compared to the early stage of disease (Fuentes et al., 2016). It is important to highlight here that 57.5% of our patients were diagnosed with CAT within the first 6 months after cancer diagnosis with 39.1% of them were diagnosed at cancer diagnosis. Countless evidence has shown that most of CAT events will occur within the first year of cancer diagnosis (Fuentes et al., 2016). 

The main goal of our real-world trials was to obtain estimates of the CAT recurrence rates in patients with cancer treated with rivaroxaban or standard of care (LMWH/fondaparinux). Our study showed there is a non-significant higher rate of recurrence in standard of care compared to rivaroxaban. This similar rate of recurrence between both arms is concordance with the current available literature (Young et al., 2018; Faqah et al., 2020; Chen et al., 2021). It is worth mentioning that the rate of recurrence in our study was higher with what has been postulated in the previous literature. In our study, the rate of recurrence in patients receiving rivaroxaban was 39% or nearly 1/3 of the population. This is high compared to 3.9% in SELECT D trial and 14.4% in the Taiwan study (Young et al., 2018; Chen et al., 2021). This may be due to our small population as compared to other studies. In SELECT D, 406 patients were recruited, and 529 patients were recruited in a Taiwan study (Young et al., 2018; Chen et al., 2021). However, this finding is alarming and warrants a further investigation.

In our study, most of the cases of recurrent PE and/or DVT in both arms were incidental, related to computed tomography (CT) imaging for tumour status, repeated CT angiography to assess PE or repeated Doppler ultrasound to assess DVT status and helping in clinical decision regarding duration of anticoagulant. It is important to note that 40% of our population requires more than 6-month anticoagulant and 26% of them need to be prescribed anticoagulant beyond 12-months. This real-world finding is important since most of studies only follows up with patients up to 6 months with little to no knowledge of the outcomes beyond that (Young et al., 2018; Raskob et al., 2018). This finding is in line with the recommendation of certain guidelines for prolonged or lifelong anticoagulation in certain populations (NCCN Guidelines, 2021; Mandala et al., 2010). The use of anticoagulant beyond 6 months will yield a tremendous burden to the healthcare system in terms of managing adverse events related to it, especially in countries where resources are restricted.

The second primary outcome of our study is death within 12 months from CAT diagnosis. It is notable to point out this is the first few studies to produce data up to 12 months. Previous studies that report mortality beyond 6-month were the XALIA trial in 2015 (Ageno et al., 2014). We observed a non-significant higher mortality rate in the rivaroxaban group compared to standard of care (31.7% vs. 20.5%, p=0.255). This finding will give some new evidence to the world of cancer especially in the field of CAT. We postulate that, this may be due to a higher number of colorectal cancers (26.8), PE incidence (36.6%) and most of them were diagnosed during the first 6 months after cancer diagnosis (61%) in rivaroxaban group. Study has shown that, cancer patient diagnosed with PE were associated with an increased risk of death (Alotaibi et al., 2018). Based on the latest Malaysia National Cancer Registry 2020, colorectal cancer is the second most common cancer that has a high mortality rate (Azizah et al., 2019). Thus, it is not surprised that, the mortality rate is higher in this arm. 

On that note, our second most important secondary outcome is bleeding. In this study we report the composite of major and minor bleeding. We observed statistically non-significant higher incidence of composite outcome in standard of care arm compared to rivaroxaban (17.9% vs. 12.2%, p=0.471). It is worth mentioning that the trends of bleeding seem to be inconsistent across the major trials (Raskob et al., 2018; Büller et al., 2012). The major bleeding in this study is comparable with the latest head-to-head study comparing DOACs with LMWH (Young et al., 2018; Agnelli et al., 2020). Even though small in sample size, our study produced consistent result with previous evidence (Young et al., 2018; Faqah et al., 2020; Chen et al., 2021; Raskob et al., 2018; Büller et al., 2012; Agnelli et al., 2020). Thus, it shown that rivaroxaban would not produce more harm compared to the current standard of care with either enoxaparin or fondaparinux in agreement with previous reported studies. 

Being a single centre study with small population provides some new information for the real-world situations. It fills the gap in the current literature from the larger RCT (Young et al., 2018; Raskob et al., 2018; Agnelli et al., 2020). The real-world experience gives more relevant evidence to patients and oncologist and helps them make their clinical judgment. We believe that rivaroxaban has a promising future in the land of CAT. This small but rather important evidence should cautiously be interpreted before applying in routine clinical practice. Besides that, rivaroxaban pharmacokinetics and pharmacodynamics should be taken into consideration before discussing the option with patients.

In conclusion , the result of this small but important real-world evidence proofs that rivaroxaban is an effective and safe alternative to standard of care for CAT in Malaysia’s cancer population. Rivaroxaban reduces the rate of recurrence CAT compared with LMWH/fondaparinux with a favourable bleeding rate. The convenient route of administration makes rivaroxaban a compelling alternative. It is important to note that rivaroxaban is metabolised by CYP450 isoenzymes specifically CYP3A4 and CYP2J2. Hence treatment initiation should be guided by drug-drug interaction with currently available drug especially oncolytics agents. At the end of the day, patient cantered clinical decision should play an important role with regard to the benefits and risk of the treatment alternatives. Further study with larger cohort of patients is needed to make the study result more generalizable to a larger audience. 

## Author Contribution Statement

All authors contributed to the study conception and design. Material preparation, data collection and analysis were performed by [Azmi-Nor-Mohd-Farez Ahmat], [Sharifa-Ezat Wan-Puteh], and [Suhana Yusak]. The first draft of the manuscript was written by [Azmi-Nor-Mohd-Farez Ahmat] and all authors commented on previous version of the manuscript. All authors read and approved the final manuscript. We confirm that all authors listed have contributed significantly to the work, have read the manuscript, attest to validity and legitimacy of the data and its interpretation, and agree to its submission to this journal.

## Declarations

We confirm that the manuscript is the authors’ original work unless appropriately cited. We also confirm that the manuscript has not received prior publication and is not under consideration for publication elsewhere.

## Availability of data and material

All date material is available upon appropriate request to corresponding author.

## Ethics approval

The study obtained ethical approval from Universiti Kebangsaan Malaysia Research Ethical Committee (UMREC-FF-2019-474) and Ministry of Health Medical Research Ethical Committee (MREC-NMRR-19-565-45643).

## Conflict of interest

We confirm that all authors of the manuscript have no conflict of interest to declare.
